# Supplementary material for: Venomix: a simple bioinformatic pipeline for identifying and characterizing toxin gene candidates from transcriptomic data
Source: PeerJ. 2018 Jul 31;6:e5361. doi: 10.7717/peerj.5361 (PMC6074769; doi:10.7717/peerj.5361)
Supplement: Supplemental Information 3 [file peerj-06-5361-s003.gz › FinalOutput_E-6/Kunitz-type_serine_protease_inhibitor_homolog_alpha-dendrotoxin_3/finaltree.pdf]

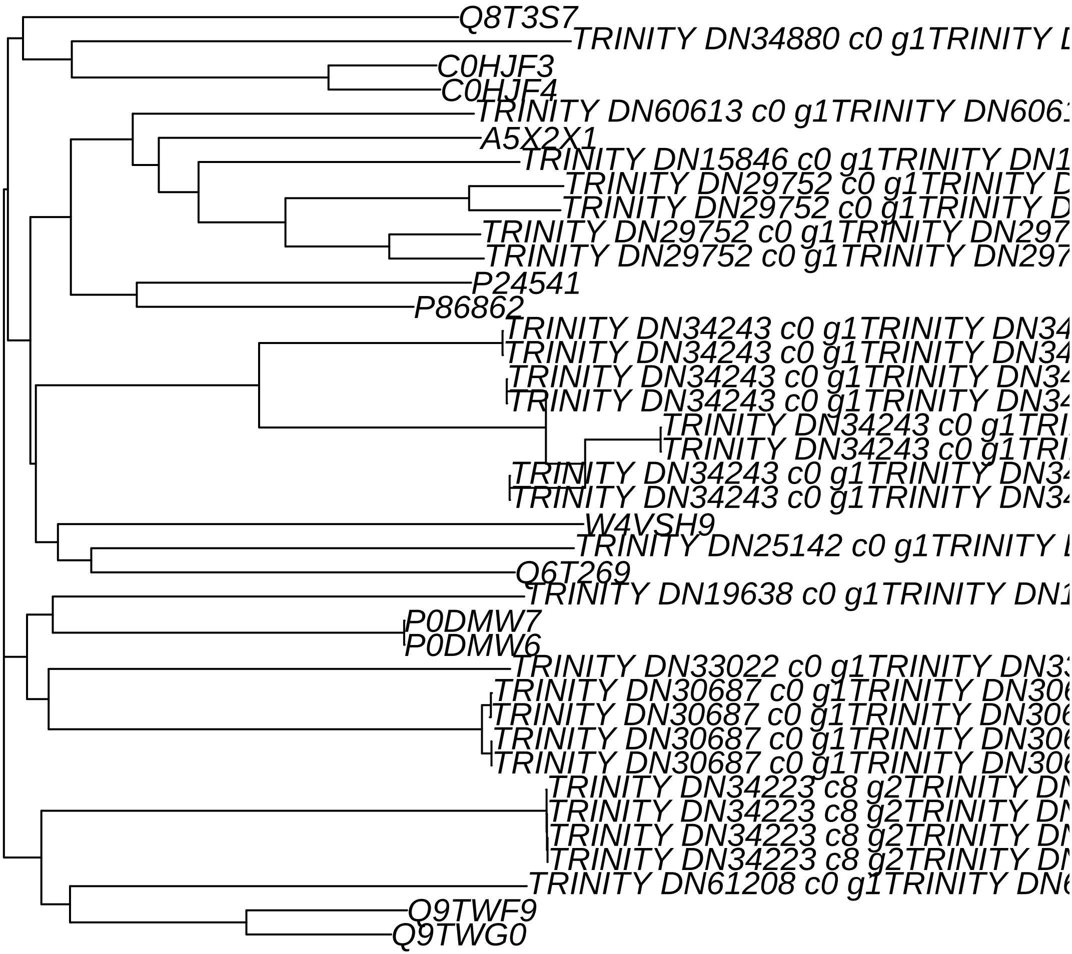
Q8T3S7
TRINITY DN34880 c0 g1TRINITY D
C0HJF3
C0HJF4
TRINITY DN60613 c0 g1TRINITY DN606
A5X2X1
TRINITY DN15846 c0 g1TRINITY DN1
TRINITY DN29752 c0 g1TRINITY D
TRINITY DN29752 c0 g1TRINITY D
TRINITY DN29752 c0 g1TRINITY DN297
TRINITY DN29752 c0 g1TRINITY DN297
P24541
P86862
TRINITY DN34243 c0 g1TRINITY DN34
TRINITY DN34243 c0 g1TRINITY DN34
TRINITY DN34243 c0 g1TRINITY DN34
TRINITY DN34243 c0 g1TRINITY DN34
TRINITY DN34243 c0 g1TRI
TRINITY DN34243 c0 g1TRI
TRINITY DN34243 c0 g1TRINITY DN34
TRINITY DN34243 c0 g1TRINITY DN34
W4VSH9
TRINITY DN25142 c0 g1TRINITY D
Q6T269
TRINITY DN19638 c0 g1TRINITY DN1
P0DMW7
P0DMW6
TRINITY DN33022 c0 g1TRINITY DN33
TRINITY DN30687 c0 g1TRINITY DN306
TRINITY DN30687 c0 g1TRINITY DN306
TRINITY DN30687 c0 g1TRINITY DN306
TRINITY DN30687 c0 g1TRINITY DN306
TRINITY DN34223 c8 g2TRINITY DN
TRINITY DN34223 c8 g2TRINITY DN
TRINITY DN34223 c8 g2TRINITY DN
TRINITY DN34223 c8 g2TRINITY DN
TRINITY DN61208 c0 g1TRINITY DN6
Q9TWF9
Q9TWG0
